# Perioperative Glutamine Supplementation May Restore Atrophy of Psoas Muscles in Gastric Adenocarcinoma Patients Undergoing Gastrectomy

**DOI:** 10.3390/nu16142301

**Published:** 2024-07-17

**Authors:** Jin-Ming Wu, Hsing-Hua Tsai, Shang-Ming Tseng, Kao-Lang Liu, Ming-Tsan Lin

**Affiliations:** 1 Department of Surgery, National Taiwan University Hospital, National Taiwan University College of Medicine, Taipei 100, Taiwan; wujm0531@ntu.edu.tw (J.-M.W.); serena751004@gmail.com (H.-H.T.); 2 Department of Surgery, National Taiwan University Hospital Hsin-Chu Branch, Hsin-Chu County 300, Taiwan; 3 Department of Surgery, National Taiwan University Hospital Jin-Shan Branch, New Taipei City 208, Taiwan; theshangming@gmail.com; 4 Department of Medical Imaging, National Taiwan University Cancer Center, Taipei 106, Taiwan; kaolangliu@gmail.com; 5 Department of Medical Imaging, National Taiwan University Hospital, National Taiwan University College of Medicine, Taipei 100, Taiwan

**Keywords:** gastric cancer, cachexia, psoas muscle, glutamine

## Abstract

Background: Sarcopenia, characterized by degenerative skeletal muscle loss, is increasingly linked to poor surgical outcomes. Glutamine, an immune-modulating formula, may stimulate muscle protein synthesis and inhibit degradation. We used the psoas major muscle area (PMMA) at the third lumbar vertebra, normalized for height (PMMA index), as a skeletal muscle indicator. This study investigates whether perioperative glutamine supplementation mitigates psoas muscle atrophy. Methods: We enrolled gastric adenocarcinoma (GA) patients undergoing gastrectomy. Computed tomography assessed the psoas muscle short axis. Muscle atrophy was estimated by changes between preoperative and three-month post-gastrectomy scans. Perioperative glutamine supplementation (PGS) comprised five-day parenteral plus one-month oral use. Propensity score matching minimized potential bias. A linear regression model predicted the association. Results: Of 516 patients analyzed (2016–2019), 100 (19.4%) received PGS. After propensity score matching, each group contained 97 cases. The PGS group showed a significantly higher median PMMA index change than the non-PGS group (0.3 vs. −0.3 cm^2^/m^2^, *p* = 0.004). Multivariate analysis revealed that PGS was significantly associated with increased PMMA index (coefficient = 0.60; 95% CI: 0.19–1.01; *p* = 0.005). Conclusions: PGS may help restore psoas muscle atrophy in GA patients undergoing gastrectomy. The underlying mechanisms likely relate to glutamine’s role in protein metabolism and immune function. Further studies are needed to elucidate these mechanisms fully.

## 1. Introduction

Gastric adenocarcinoma (GA) is one of the most common foregut cancers worldwide and is often diagnosed at advanced stages unless routine endoscopy is carried out [1]. Dysphagia and loss of body weight are often encountered in GA patients with advanced stages of cancer. Radical gastrectomy plus lymphadenectomy is considered as the only potentially curative treatment. However, it is accompanied by a high rate of morbidity and mortality, especially in GA patients with major comorbidities, poor physical activity, or malnutrition [2,3,4]. Notably, in the postoperative period, GA patients suffer from the deterioration of strength in skeletal muscles and poor quality of life due to the complex reconstruction of the gastrointestinal tract and gastrointestinal tract sequelae [5]. Since physical activity is associated with outcomes in GA cases, its improvement is crucial.

Sarcopenia, previously known as muscle weakness [6] or cancer-related cachexia [7], is measured as the skeletal muscle index (SMI), ≤55 cm^2^/m^2^ for men and ≤39 cm^2^/m^2^ for women [8]. SMI is strongly correlated with the cross-sectional area of the psoas major muscle (PMMA) at the third lumbar vertebra (L3), which provides an easier method to assess the severity of sarcopenia among GA patients [9]. This parameter is often measured using abdominal computed tomography (CT). Recently, there are increasing studies investigating the association between sarcopenia and outcomes, and this correlation is highlighted in surgical patients for foregut cancer [6,10].

Glutamine is the most abundant non-essential amino acid in humans; it is depleted under hypermetabolic and hyper catabolic conditions, such as severe illness or major surgery [11,12]. Since glutamine is involved in diverse processes, including protein metabolism [13] and immune system modulation [14], its insufficiency results in negative nitrogen balance, causing significant dysfunction of the gut barrier and wound healing [15]. Several studies support that glutamine supplementation is beneficial for the recovery of patients from critical illness as well as chemotherapy-related side effects [16,17,18]. However, the impact of glutamine supplementation on sarcopenia was inconclusive [19].

Therefore, this study was conducted to evaluate whether perioperative use of glutamine aids in improving sarcopenia in GA patients undergoing gastrectomy. We hypothesized that glutamine might play a role in improving sarcopenia from the perspective of muscle quantity. As CT is routinely performed in surgical GA patients to check for cancer recurrence, we used PMMA to assess the severity of sarcopenia from the perspective of muscle quantity. The primary endpoint was the perioperative change in PMMA.

## 2. Materials and Methods

### 2.1. Study Population

Patients aged ≥20 years with GA receiving gastrectomy were eligible for enrollment in the present retrospective observational study that was approved by the local ethics committee (National University Hospital; 201909033RIND). The study period was from January 2016 to June 2019. We collected data on the clinical demographics, surgical procedures, and cancer stages of the patients [20].

The weighted Charlson comorbidity index (CCI) score was calculated to represent the severity of comorbidity for each case [21]. All postoperative complications were retrieved from the medical records, and the major postoperative complications were defined as Clavien grade ≥ 3 [22].

**Figure 1:**
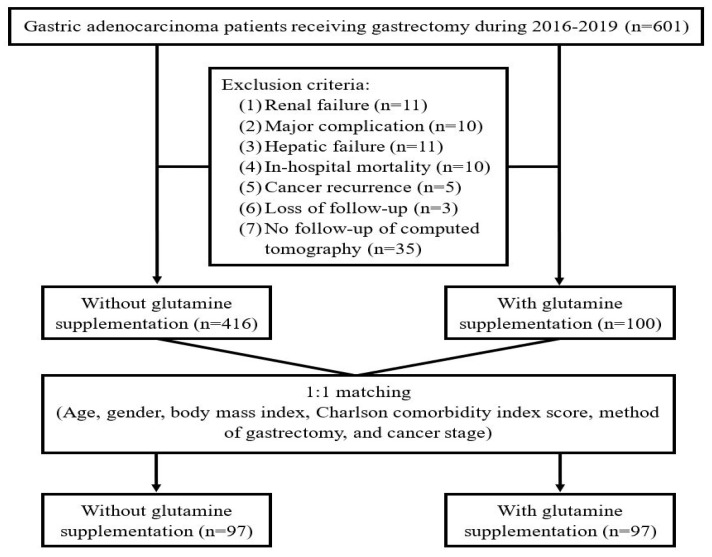
Flow diagram for the inclusion and exclusion criteria in this study.

A total of 601 patients undergoing gastrectomy for GA were enrolled in the study. After excluding renal failure (*n* = 11), hepatic failure (*n* = 11), major complications (*n* = 10), in-hospital mortality (*n* = 10), cancer recurrence (*n* = 5), loss to follow-up (*n* = 3), and no follow-up of CT (*n* = 35), 516 subjects were included for the analysis (Figure 1).

### 2.2. Measurement of Psoas Major Muscle

One trained study assessor who was blinded to the CT data analyzed the axial consecutive images at the top of the iliac crest (IC) level at the preoperative period and 3 months postoperatively (POM). The real slice level was the CT image in which IC had only vanished, which was well matched with the L3 level [23]. PMMA was measured by using an image viewer software, “Radiant DICOM Viewer 4.2” (Medixant, Poznan, Poland), on a desktop computer screen.

All measurements were performed by the segmentation of the skeletal muscle based on the Hounsfield Unit (HU) thresholds (−30 to +150 HU) to exclude vasculature or soft tissues. Furthermore, PMMA was normalized for height (PMMA index). The primary endpoint was a change in the PMMA index (PMMA index at 3 POM minus PMMA index at the preoperative period).

### 2.3. Perioperative Glutamine Supplementation

The study group included patients with both 5-day inpatient supplementation of parenteral glutamine (Dipeptiven^®^; Fresenuis Kabi, Hamburg, Germany; 100 mL per bottle with 20 g N(2)-L-alanyl-L-glutamine) and outpatient oral glutamine (Sympt-X, Plano, TX, USA; 10 g glutamine) thrice daily for 28 days.

### 2.4. Statistical Analysis

All variables were calculated as percentages, and continuous variables were summarized as the median and interquartile range. We employed Student’s *t*-test to compare the means of two continuous variables. In addition, the Chi-square test and Fisher’s exact test were used to test the correlation among the categorical variables.

We used propensity score (PS) matching to diminish the disparities of confounding factors in the background characteristics between the two groups [24]. By logistic regression, the PS model with a matching ratio of 1:1 was constructed with the presence or absence of perioperative glutamine supplementation (PGS) serving as the dependent variable. Clinicodemographic (i.e., age, gender, and body mass index), CCI score, cancer stage, type of gastrectomy, whether receiving neoadjuvant chemotherapy, and their interactions served as the independent variables [25].

The independent effects of study variables on the PMMA index were determined using linear regression models with inclusive models that included all clinicodemographic and pathological data. The results were presented as ORs with 95% confidence intervals (CIs). Statistical analyses were performed using the Stata software (version 13.1; StataCorp, College Station, TX, USA). A two-tailed *p* < 0.05 was considered to indicate statistical significance.

## 3. Results

Of the 516 patients, 100 (19.4%) received glutamine supplementation. Table 1 demonstrates the clinicopathological and surgical differences between the patients without glutamine supplementation and with glutamine supplementation. The median age of the patients with glutamine supplementation (glutamine group) was higher than that of the patients without glutamine supplementation (non-glutamine group; 68.3 vs. 64.3; *p* = 0.040). Further, the methods of gastrectomy between the two groups were statistically different (*p* < 0.001).

**Figure 2:**
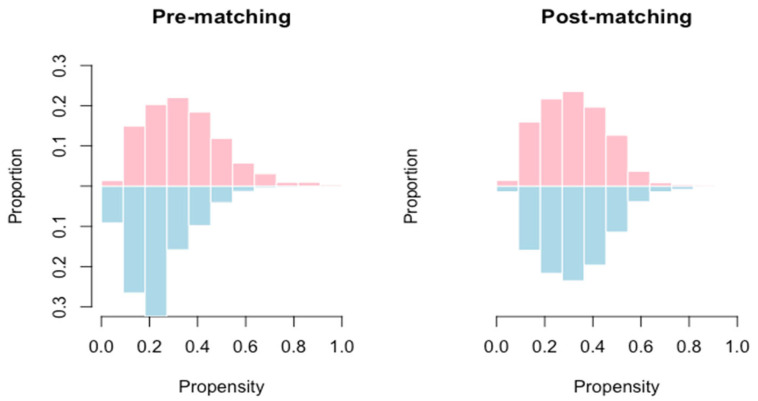
Distribution of the estimated propensity score for receiving perioperative parenteral glutamine supplementation between two groups before and after matching.

After matching the PS, 97 cases were left in each group. Figure 2 shows the distribution of the estimated PS for receiving PGS before and after matching. Further, Table 2 presents a comparison between the glutamine group and the non-glutamine group. The differences in clinical demographics and surgical procedures and cancer stages were not statistically significant.

### Measurement of Area of Psoas Major Muscle

Among the matched cohorts, the median value of the preoperative PMMA index in glutamine and non-glutamine groups was 4.9 cm^2^/m^2^ and 5.0 cm^2^/m^2^ (*p* = 0.850), respectively. The median value of the 3-month postoperative PMMA index in glutamine and non-glutamine groups was 5.6 cm^2^/m^2^ and 5.0 cm^2^/m^2^ (*p* < 0.001), respectively. The median change in the PMMA index in the glutamine group was significantly higher than that in the non-glutamine group (0.3 vs. −0.3 cm^2^/m^2^, *p* = 0.004). The adjusted model (Table 3) showed that glutamine supplementation was significantly associated with increased PMMA index (coefficient = 0.60; 95% CI: 0.19–1.01; *p* = 0.005). On the contrary, patients aged > 75 years (coefficient = −1.05; 95% CI: −1.48 to −0.62; *p* < 0.001) and between 66 and 75 years (coefficient = −0.12 per one kg/m^2^ increment; 95% CI: −0.17 to −0.07; *p* < 0.001) showed a negative correlation with PMMA index in comparison with age ≤ 65 years.

## 4. Discussion

Patients with advanced gastric cancer often suffer from malnutrition and sarcopenia. Their condition further deteriorates after radical gastrectomy plus lymphadenectomy due to surgical stress and gastrointestinal sequelae. Glutamine has been proved to be a vital nutrient associated with tissue repair, protein metabolism, and maintenance of cell function [26,27,28]. The present study showed that PGS restored the early postoperative atrophy of the psoas major muscle in GA patients subjected to gastrectomy. Our study serves as a pilot study with a large sample size, elucidating the clinical impacts of PGS on surgical GA subjects.

The mechanism by which PGS contributes to reduced atrophy in the psoas major muscle may be related to the protein metabolism in muscles, since glutamine is an important nutrient and has a role in anabolic processes [29,30]. Glutamine, one of the most abundant non-essential amino acids in humans, is also present in high amounts in skeletal muscles. It can become conditionally essential (when the synthesis rate of glutamine is not enough to meet the demands) in cancer patients or hyper catabolic conditions, such as major surgery [31]. The supplementation of glutamine restores its deficiency in GA patients and attenuates sarcopenia by increasing protein synthesis in muscles and thus improving the nitrogen balance [32]. The results of our study conform with the previous literature, wherein PGS improved fat-free mass and contributed to maintaining lean body mass in surgical patients with head and neck malignancy [33]. Nonetheless, this association was not noted in patients with muscular dystrophy or resistance-trained subjects [34]. The key factor to elucidate this inconclusive finding may be whether the case had glutamine deficiency or not. However, this assertion needs to be investigated further.

In the current study, older age was significantly associated with early postoperative atrophy of the psoas major muscle, while very old patients (age > 75 years) showed a higher decrease in the area of the psoas muscles. Muscular aging is a complex physiological phenomenon, involving a multifactorial origin in habits/lifestyle, a decline in mitochondrial function in skeletal muscle, and hormonal balance [35], and this decline upon reaching a pathological level contributes to adverse outcomes [36]. Notably, our study population was also diagnosed with cancer, which deteriorated aforementioned muscle wasting. The mechanism may be associated with cancer cachexia, a complex metabolic syndrome leading to systemic inflammation and impairment of the immune system [37]. Recently, due to the increasing number of cancer treatments among the elderly, it is imperative to diagnose cachexia early and implement effective interventions for improving the outcomes. Surgeons should monitor and evaluate oncological outcomes and sarcopenia through serial CT scans.

The present study also has some limitations. Firstly, this study has a retrospective design and was conducted in a single academic institute. Therefore, the findings may not be applied widely. The external validity of the study requires randomized clinical trials. Secondly, our study did not measure the data related to nutritional intake and physical activity, which might also affect muscle mass. Thirdly, we evaluated the association between PGS and short-term changes in the psoas muscle. The long-term effects of the treatment should also be considered and addressed in future studies. Fourthly, some studies questioned that the psoas muscular index has limitations in estimating skeletal muscle mass, which may interfere with the study population, ethnic differences, or non-muscular factors like hydration and edema [38]. These limitations highlight the need for caution when using the psoas muscular index as a sole indicator of muscle mass or health status, particularly across diverse populations or in complex clinical scenarios. Fifthly, the standard definition of sarcopenia has evolved over time, but the most widely accepted current definition should take muscle quantity, muscle strength, and physical performance into consideration. In this study, we only calculate the muscle quantity, which may cause overestimation or underestimation of sarcopenia.

## 5. Conclusions

PGS may restore the atrophy of the psoas muscle among GA patients undergoing gastrectomy. The mechanism by which this effect is achieved may be associated with protein metabolism and immune function, as glutamine plays a key role in both these processes. Further studies should be designed to validate this hypothesis and uncover the molecular mechanisms.

## Figures and Tables

**Table 1:** Comparison of clinicopathological variables between gastric cancer patients undergoing gastrectomy that did or did not receive perioperative glutamine supplementation before matching.

Factor (Number or Median [IQR])	Glutamine Supplementation		*p* Value
	No (*n* = 416)	Yes (*n* = 100)	
Age	64.3 (56.5, 74.8)	68.3 (59.3, 74.8)	0.04
Gender			0.19
Female	178 (42.8%)	50 (50.0%)	
Male	238 (57.2%)	50 (50.0%)	
Body mass index	23.1 (20.5, 25.3)	23.6 (21.5, 26.5)	0.076
Charlson comorbidity index			0.078
≤2	256 (61.5%)	71 (71.0%)	
>2	160 (38.5%)	29 (29.0%)	
Cancer stage			0.7
I	75 (18.0%)	19 (19.0%)	
II	150 (36.1%)	32 (32.0%)	
III	169 (40.6%)	41 (41.0%)	
IV	22 (5.3%)	8 (8.0%)	
Method of gastrectomy			<0.001
Open distal gastrectomy	206 (49.5%)	71 (71.0%)	
Laparoscopic distal gastrectomy	67 (16.1%)	19 (19.0%)	
Open total gastrectomy	143 (34.4%)	10 (10.0%)	
Adjuvant chemotherapy	88 (21.1%)	21 (21.0%)	0.88

IQR: interquartile range.

**Table 2:** Comparison of clinicopathological variables between gastric cancer patients undergoing gastrectomy that did or did not receive perioperative glutamine supplementation after matching.

Factor (Number or Median [IQR])	Glutamine Supplementation		*p* Value
	No (*n* = 97)	Yes (*n* = 97)	
Age	67.4 (58.4, 73.7)	68.3 (59.2, 74.4)	0.57
Gender			0.89
Female	46 (47%)	47 (48%)	
Male	51 (53%)	50 (52%)	
Body mass index	23.5 (21.6, 25.9)	23.6 (21.6, 26.4)	0.93
Charlson comorbidity index			0.52
≤2	72 (74%)	68 (70%)	
>2	25 (26%)	29 (30%)	
Cancer stage			0.64
I	13 (13%)	19 (20%)	
II	36 (37%)	30 (31%)	
III	40 (41%)	40 (41%)	
IV	8 (8%)	8 (8%)	
Adjuvant chemotherapy	20 (20.6%)	20 (20.6%)	0.999
Method of gastrectomy			0.96
Open distal gastrectomy	68 (70%)	68 (70%)	
Laparoscopic distal gastrectomy	20 (21%)	19 (20%)	
Open total gastrectomy	9 (9%)	10 (10%)	
Measurements of psoas major muscle			
Pre-operative area of psoas major muscle (cm^2^/m^2^)	5.0 (4.5, 5.8)	4.9 (4.1, 6.2)	0.85
3-month postoperative area of psoas major muscle (cm^2^/m^2^)	5.0 (4.4, 5.6)	5.6 (5.0, 6.2)	<0.001
Change in area of psoas major muscle (cm^2^/m^2^)	−0.3 (−0.8, 0.5)	0.3 (−0.4, 1.6)	0.004

IQR: interquartile range.

**Table 3:** Multivariate analysis to predict the change in the cross-sectional area of the psoas major muscle index in the propensity score model.

Variables	Coefficients	95% Confident Interval		*p* Value
		Lower limit	Upper limit	
Age category (ref: ≤65 years)				
66–75 years	−0.12	−0.17	−0.07	<0.001
>75 years	−1.04	−1.48	−0.62	<0.001
Male gender (ref: female)	0.09	−0.40	0.71	0.576
Body mass index	0.39	−0.10	0.88	0.121
Charlson comorbidity index scores > 2 (ref: ≤2)	0.05	−0.42	0.52	0.828
Cancer stage (ref: stage I)				
Stage II	0.58	−0.07	1.23	0.081
Stage III	0.24	−0.38	0.86	0.443
Stage IV	0.30	−0.64	1.23	0.532
Method of gastrectomy (ref: open distal gastrectomy)				
Open total gastrectomy	−0.14	−0.67	0.39	0.596
Laparoscopic distal gastrectomy	0.30	−0.43	1.05	0.413
Glutamine supplementation	0.60	0.19	1.01	0.005

## Data Availability

Data may be available if requested to the corresponding author because of legal reason.
